# Right-Sided Intrarenal Splenosis Mimicking a Renal Carcinoma

**DOI:** 10.1100/tsw.2006.380

**Published:** 2006-06-07

**Authors:** Jay B. Page, Dean L. Lenz, Carson Wong

**Affiliations:** ^1^ Department of Urology, University of Oklahoma Health Sciences Center, Oklahoma City, OK, USA; ^2^ Division of Urology, Pennsylvania State University, Milton S. Hershey Medical Center, Hershey, PA, USA

**Keywords:** splenosis, splenorenal fusion, renal mass, renal carcinoma

## Abstract

We describe a patient who underwent nephrectomy for an enhancing right renal mass that was subsequently pathologically confirmed as right renal splenosis. Since renal splenosis is quite rare and has previously been reported only in the left kidney, we did not consider splenosis in our differential diagnosis during the evaluation of the renal mass. Magnetic resonance imaging, as well as radionucleotide scan using 99mTc-labelled red blood cells, has been utilized for identifying ectopic splenic tissue. An elevated index of suspicion must be present in patients with a history of splenectomy or traumatic splenic rupture to avoid undue nephrectomy.

## INTRODUCTION

The presence of benign splenic tissue within the renal parenchyma, or renal splenic heterotopia, is very rare and can be radiographically misinterpreted as a primary neoplasm of the kidney. When occurring in a patient with an intact spleen, this entity is known as splenorenal fusion. In a patient who has undergone splenectomy, its occurrence is referred to as splenosis. Herein, we describe an enhancing right renal mass found to represent right lateralized renal splenosis.

## CASE REPORT

An asymptomatic, 55-year-old male was evaluated for an incidentally detected right renal mass. Past medical history included splenectomy for idiopathic thrombocytopenic purpura. The physical examination was unremarkable. Urinalysis, complete blood count, and serum chemistries were within normal limits. Computed tomography (CT) of the abdomen and pelvis without and with intravenous contrast demonstrated a 4- × 3-cm solid enhancing lesion within the right kidney (Fig. 1).

Following a negative metastatic evaluation, the risks and benefits of radical nephrectomy, partial nephrectomy, radiofrequency ablation, cryotherapy, and serial surveillance were discussed. Given the proximity of the mass near the collecting system on CT, the patient elected for a right laparoscopic radical nephrectromy. The postoperative course was unremarkable. Pathologic examination of the specimen revealed mature splenic tissue within the renal parenchyma without evidence of a primary renal malignancy (Fig. 2).

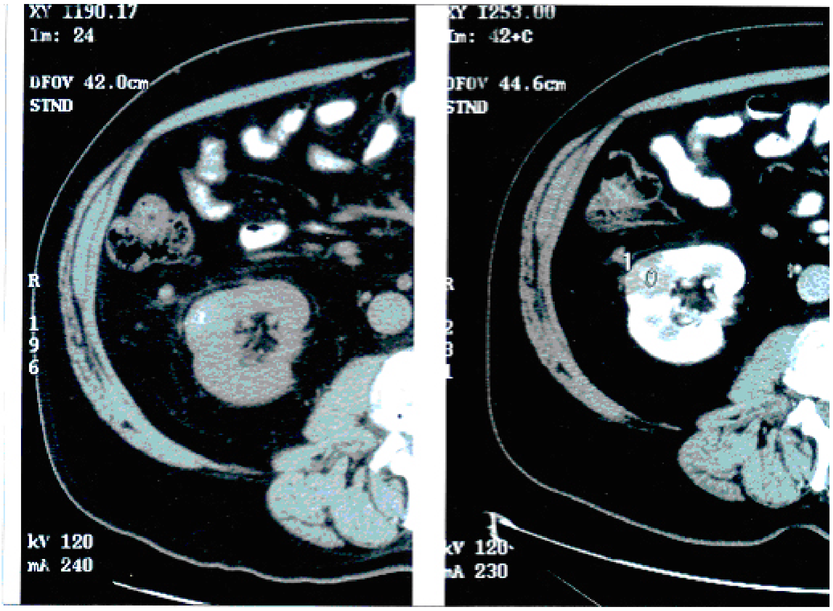

**FIGURE 1.** CT of the right kidney pre- and postintravenous contrast, demonstrating a solid enhancing lesion (30.94 to 91.32 Hounsfeld Units).

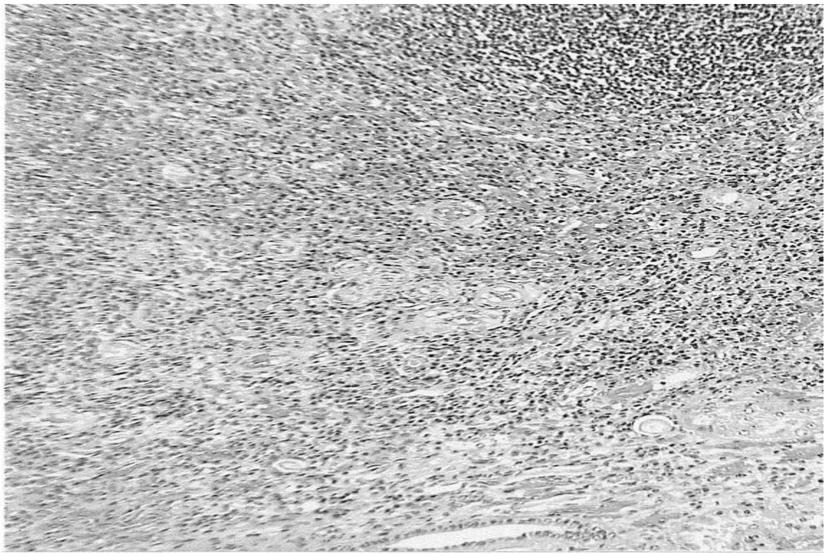

**FIGURE 2.** Hematoxylin and Eosin stain of right kidney, demonstrating normal renal parenchyma with elements of splenic tissue (150×).

## DISCUSSION

Ectopic splenic tissue occurs in up to 30% of the population[1]. Accessory spleens result from failed fusion of the mesenchyme during splenic development. The most common location for accessory splenic tissue is the splenic hilum and its immediately adjacent areas.

Splenovisceral fusion, the fusion of heterotopic splenic tissue with normal visceral tissue, is a rare entity that occurs with a normal spleen in its orthotopic position. Reported sites of fusion include the gonads, liver, retroperitoneum, and kidney (three left kidney, one right)[2]. One theory for splenoviseral fusion includes "transcoelomic adhesion", which involves fusion of the developing spleen and mesonephric ridge[2,4]. A second theory describes migration of splenic tissue via the caudal limiting fold, a portion of the developing diaphragm[1,4]. These theories, however, fail to explain the existence of right-sided splenorenal fusion adequately. Rosenthal et al. suggest that the holonephric theory of renal development, the direct migration of splenic tissue to the retroperitoneum, could account for a right-sided occurrence[1].Splenosis refers to implantation of splenic tissue following splenectomy. Most cases reported are due to traumatic splenic injury, although elective splenectomy for hematological disease may also lead to ectopic splenic tissue[5]. Splenosis can occur in multiple locations including the small bowel serosa, omentum, peritoneum, and diaphragm. A review of the literature revealed five cases of left-sided renal splenosis and none on the right[2]. One case of right-sided retroperitoneal splenosis has been reported in a patient with history of splenic rupture[6].

Magnetic resonance imaging (MRI) with ferumoxide has been described for differentiating splenic parenchyma from intrarenal masses[3]. The best-studied modality for detecting ectopic splenic tissue remains 99mTc-labelled erythrocytes.

The clinical significance of heterotopic splenic tissue includes the recurrence of splenic function after removal of an orthotopic spleen, misdiagnosis of fused spleen as a surgical lesion, hemorrhage, pain, or recurrence of a hematologic malignancy. Splenic heterotopia can be demonstrated using 99mTc-sulfur-colloid radionucleotide scan, which can confirm the presence of splenic tissue $\geq 2$ cm in diameter. MRI or radionucleotide scanning should be utilized in patients with a history of splenectomy or traumatic rupture to avoid the unnecessary morbidity of nephrectomy.

Right intrarenal splenosis has not been described previously, and is herein described to heighten the awareness of this condition along with possible preoperative imaging options to eschew avertable nephron loss.

## ACKNOWLEDGEMENT

We are grateful to Beverly K. Shipman for her assistance in the preparation of this manuscript.
